# Cerebrospinal fluid pro-inflammatory cytokines differentiate parkinsonian syndromes

**DOI:** 10.1186/s12974-018-1339-6

**Published:** 2018-11-03

**Authors:** C. Starhof, K. Winge, N. H. H. Heegaard, K. Skogstrand, S. Friis, A. Hejl

**Affiliations:** 1 Department of Neurology, Bispebjerg University Hospital, Bispebjerg Bakke 23, Copenhagen, Denmark; 2 Department of Neurology, Roskilde University Hospital, Roskilde, Denmark; 3 Department of Clinical Biochemistry, University of Southern Denmark, Odense, Denmark; 4 Department of Autoimmunology and Biomarkers, Statens Serum Institute, Copenhagen, Denmark; 5 Center for Neonatal Screening, Department of Congenital Disorders, Statens Serum Institute, Copenhagen, Denmark; 6 Danish Cancer Society Research Center, Danish Cancer Society, Copenhagen, Denmark; 7 Department of Public Health, Copenhagen University, Copenhagen, Denmark

**Keywords:** Parkinson’s disease, Multiple system atrophy, Progressive supranuclear palsy, Cerebrospinal fluid, Biomarkers, Cytokines, C-reactive protein

## Abstract

**Introduction:**

Neuroinflammation has been established to be part of the neuropathological changes in Parkinson’s disease (PD) and atypical parkinsonism (APD). Activated microglia play a key role in neuroinflammation by release of cytokines. Evidence of the disparity, if any, in the neuroinflammatory response between PD and APD is sparse. In this study, we investigated CSF cytokine profiles in patients with PD, multiple system atrophy (MSA), or progressive supranuclear palsy (PSP).

**Methods:**

On a sensitive electrochemiluminescence-based platform (Quickplex, Meso Scale Discovery®), we examined a panel of C-reactive protein (CRP) and eight selected cytokines, IFN-γ, IL-10, IL-18, IL-1β, IL-4, IL-6, TGF-β1, and TNF-α, among patients with PD (*n* = 46), MSA (*n* = 35), and PSP (*n* = 39) or controls (*n* = 31). Additionally, CSF total tau protein levels were measured as a marker of nonspecific neurodegeneration for correlation estimates.

**Results:**

CRP and the pro-inflammatory cytokines TNF-α, IL-1β, and Il-6 were statistically significantly elevated in MSA and PSP patients compared to PD patients but not compared to control patients. No analytes differed statistically significantly between MSA and PSP patients. The best diagnostic discrimination, evaluated by ROC curve (AUC 0.77, *p* = 007, 95% CI 0.660–0.867), between PD and MSA patients was seen for a subset of analytes: CRP, TNF-α, IL-1β, and IFN-γ.

**Conclusion:**

Among the investigated cytokines and CRP, we found a statistically significant increase of microglia-derived cytokines in MSA and PSP patients compared to PD patients.

**Electronic supplementary material:**

The online version of this article (10.1186/s12974-018-1339-6) contains supplementary material, which is available to authorized users.

## Background

Neuroinflammation co-exists with neurodegeneration in Parkinson’s disease (PD), atypical parkinsonism (APD), and neurodegenerative disorders in general [1]. PD is neuropathologically characterized by a progressive loss of dopaminergic neurons in the substantia nigra [2], and postmortem studies have identified activated microglia in substantia nigra linking PD to neuroinflammatory changes [3]. In the pathogenesis of the atypical Parkinson disorders, multiple system atrophy (MSA), and progressive supranuclear palsy (PSP), microglia activity also accompanies neurodegeneration [4, 5]. Little evidence is available on whether disparity exists in neuroinflammation between PD and APD.

Microglia, the resident innate immune cells of the brain, act as macrophages and constitute 10–20% of glial cells [6] and function through a network of secreted cytokines and chemokines acting as signaling molecules upon activation. Activated microglia may take a pro-inflammatory or anti-inflammatory phenotype.

Cytokines can be detected in the cerebrospinal fluid (CSF), and previous studies have demonstrated that IL-1β and IL-6 [7], TNF-α [8], and IL-8 [9] are elevated in the CSF in PD patients compared to controls. Other studies have, however, demonstrated an opposing pattern [10], and the biomarker potential of CSF cytokines is thus unclear. To what extent the increase in cytokine levels reflects specific inflammatory mechanisms or is a result of a quantitative increase in brain pathology is unknown.

In this study, we investigated the expression of selected cytokines in CSF in PD, MSA, or PSP, compared to controls. The aim of the study was to evaluate differences in cytokine profile across PD and two APDs with differences in neuropathological hallmarks as well as evaluate the potential of cytokines to differentiate between MSA and PD (two synucleinopathies).

## Methods

### Patients

We included patients with PD, MSA, PSP, and symptomatic controls (SC; definition [11]). All patients were included consecutively from the tertiary Movement Disorders Centre at the Department of Neurology, Bispebjerg University Hospital, from October 2007 to October 2015. For inclusion, structural neuroimaging (computed tomography (CT) scan or magnetic resonance imaging of the brain) and/or a DaTSCAN as part of the diagnostic work-up was required. All diagnoses were assigned by an experienced movement disorder specialist (KW), blinded to laboratory results, and all included patients met the clinical criteria for possible or probable diagnosis of the study diseases at sampling [12–14]. After diagnostic follow-up, only samples collected from patients fulfilling the criteria for probable disease at the time of follow-up was included. Follow-up time was noted as time from sampling until death or most recent recorded clinical control.

The SCs were included through the Neurological Emergency Department, Bispebjerg University Hospital. Eligible SCs included individuals with isolated acute onset headache suspected of subarachnoid hemorrhage and individuals with subsequently normal CT scan and neurological examination, who required lumbar puncture as part of diagnostic workout, and CSF without xanthochromia.

Basic characteristics, Hoehn and Yahr (HY) score, Levodopa Equivalent Doses (LED), data on use of anti-inflammatory drugs (nonsteroidal anti-inflammatory drugs (as chronic therapy), glucocorticoids, disease-modifying anti-rheumatic drugs, biological pharmaceuticals, antimetabolites, cyclophosphamide), and Charlson Comorbidity Index scores [15] were obtained. As a proxy for the degree of nonspecific neurodegeneration, we measured total Tau (t-tau) protein in the CSF. To investigate potential leakage of the blood-brain barrier, the plasma/CSF albumin ratio was assessed. CSF samples were analyzed for routine parameters: cell count, total protein level, and hemoglobin contamination.

### Sampling

Lumbar puncture was primarily performed using an atraumatic needle (22G, Smiths Medical). CSF was collected in a polypropylene tube placed on ice. Samples were centrifuged (10 min., 2000*g*, 4 °C within 30 min) and the supernatant aliquoted in 400-μl polypropylene tubes and placed at − 80 °C within 90 min.

### Laboratory methods

A multiplex panel consisting of CRP and eight cytokines (IFN-γ, IL-10, IL-18, IL-1β, IL-4, IL-6, TGF-β1, and TNF-α) was applied. We aimed to include microglia-derived cytokines representing both pro-inflammatory and anti-inflammatory pathways. Furthermore, inflammatory markers which had previously been studied in parkinsonian syndromes were included based on a review of existing literature. The cytokines were quantified using an in-house assay and a sensitive electrochemiluminescence-based platform (Quickplex, Meso-Scale Discovery®, MD). In short, the specific antibodies were biotinylated, bound to different linkers 1–8 (Meso-Scale, Maryland), mixed together to the concentrations 10 μg/ml per antibody, added to each U-plex plate well (50 μl/well), and incubated for 1 h. After washing with washing buffer (PBS containing 0.05% Tween 20), 25 μl undiluted CSF from each sample was added, the plates were sealed and incubated with shaking for 2 h, and washed three times. The corresponding detection antibodies were sulfo-tagged using MSD Gold Sulfo-tag NHS-Ester (Meso-Scale, R91AO-2), added 25 μl to each well, conc. 0.1 μg/ml, and incubated with shaking for 2 h. Finally, the plates were washed, 150 μl 2× Read buffer T (Meso-Scale R92TC) was added per well, and immediate readings were performed using a QuickPlex reader. Concentrations were calculated with Discovery Workbench software (Meso-Scale) from calibration curves using four-parameter logistic fit. All antibodies were purchased from RnDsystems.

T-tau levels were measured in 100 μl of CSF using a fully validated enzyme-linked immunosorbent assay (ELISA) kit (Innotest®, Fujiriebio Diagnostics AB, Göteborg, Sweden) according to the manufacturer’s protocols. The plasma/CSF albumin ratio was assessed by immunonephelometry on a ProSpec BN instrument (Siemens).

### Statistical analysis

Statistical analyses were performed with SAS Enterprise Guide 7.1 and GraphPad Prism 6. Demographics and analytes were compared by Kruskal-Wallis test for medians, Pearson chi-square for proportions, or one-way ANOVA if data followed an approximative Gaussian distribution. All cytokine levels fitted a non-normal distribution. To minimize risk of confounding, a multivariate generalized linear model adjusting for age, gender, and use of any anti-inflammatory drug was fitted with log-transformed values of cytokines. Group-wise comparisons were adjusted with the Tukey correction.

Univariate correlations were performed with Spearman correlations. The Bonferroni correction was used to correct for multiple comparisons. However, in a few samples, the measured concentrations were reported under the detection limit for the assay and were noted as half of the detection limit for the analysis.

Receiver operating characteristic (ROC) curves were constructed to evaluate the biomarker potential of each investigated analyte and for the best subset (maximum 4). The best subset of analytes was identified in a logistic regression model by score selection (SAS Enterprise Guide 7.1).

## Results

The study population comprised 154 patients (70 women and 84 men). Medical history review revealed remitting multiple sclerosis (SC), ulcerative colitis (SC), and polymyalgia rheumatica (PSP) in three patients who were excluded from further analysis, leaving 151 patients for final analysis. Demographics are presented in Table 1.

**Table 1:** Demographics

	PD	MSA	PSP	Controls (SC)
*N* (male/female)^a^	30/16	13/22	25/14	15/16
Age at sampling (years), mean (SD)^b^	64.46 (11.5)	63.48 (7.8)	69.77 (5.1)	45.48 (17.7)
Disease duration at puncture (months), mean (SD)^b^	83.78 (45.9)	63.51 (23.8)	54.8 (28.4)	–
Follow-up time (months), mean (SD)^b^	11.3 (9.2)	18.2 (16.5)	18.9 (14.6)	–
H + Y stages, median, IQR	2 (2)	3 (1)	3 (1)	–
Comorbidity, Charlson score (age-adjusted)	0.81	0.63	1.10	0.60
Levodopa Dose Equivalents, mg, SD^b^	596.8 (382.6)	712.28 (552.7)	324.2 (369.0)	–

^a^Kruskal-Wallis or one-way ANOVA *p* < 0.05, Bonferroni corrected

^b^*p* < 0.05 chi-square

Groups differed significantly in age, gender, and disease duration at sampling. SCs were younger than disease groups, and PD patients had longer disease duration. Except for IL-18 and TGF-β1, all investigated cytokines correlated positively with age, but no correlation with disease duration or LED was observed (Additional file 1: Table S1). With the exception of IL-4, t-tau did not correlate statistically significantly with analytes. Disease duration and HY did not correlate (*r* = − 0.07, *p* = 0.42) in any groups (PD *r* = 0.25, *p* = 0.09; MSA *r* = 0.12, *p* = 0.48; PSP *r* = 0.15, *p* = 0.33).

### CSF concentrations of analytes

Overall, we found statistically significantly higher concentrations of CRP, TNF-α, IL-1β, IL-4, and IL-6 in MSA and PSP compared to PD. In addition, the level of Il-10 was statistically significantly different between PSP and PD. INF- γ, IL-18, and TGF- β1 did not differ significantly between the patient groups.

**Fig. 1:**
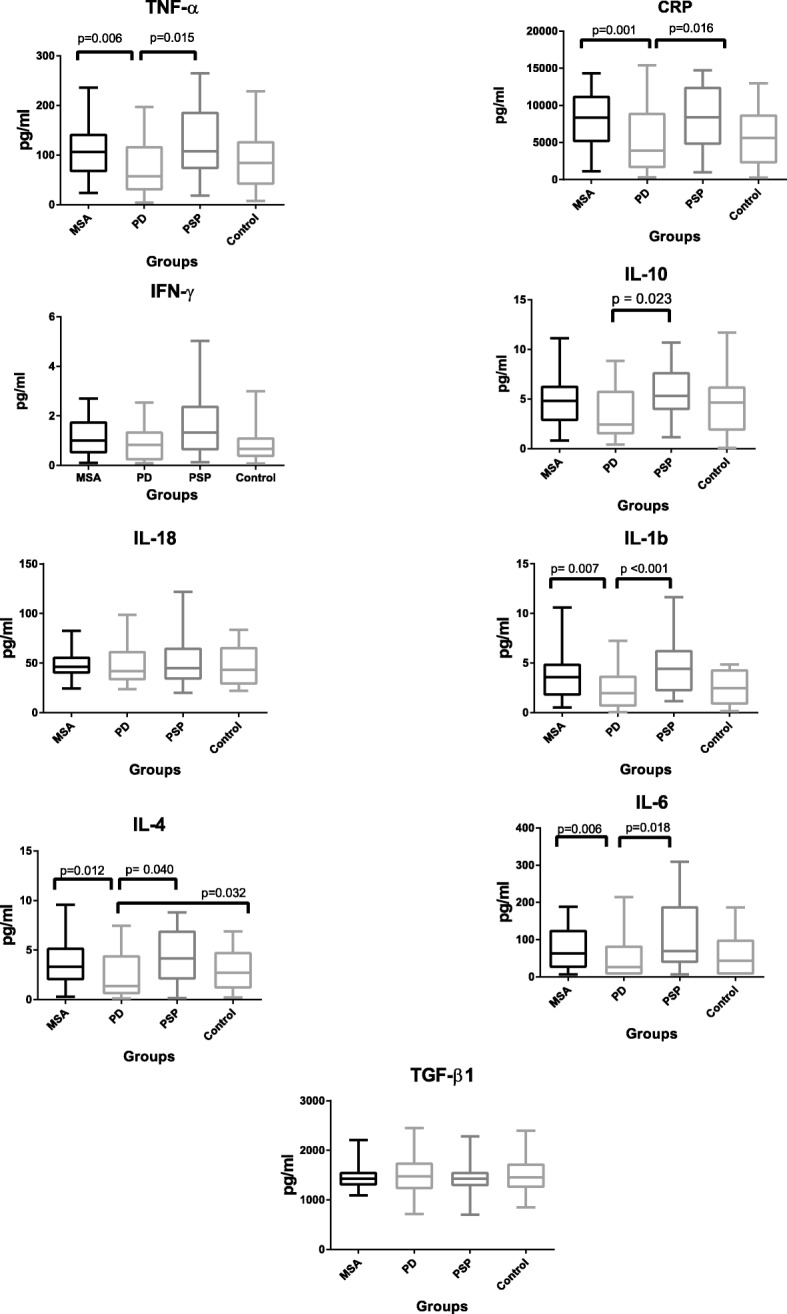
Box plots of CRP and eight selected cytokines plotted in diagnostic groups. The plots are presented without outliers. *p* values are adjusted for age, gender, and use of anti-inflammatory medication

In addition, except for IL-4, no analytes exhibited a statistically significantly different distribution between PD and SC. Likewise, no analyte differed statistically significantly between SC and APD. Box plots are presented in Fig. 1.

The albumin CSF/plasma ratio was similar in disease groups and, as expected, somewhat reduced among the SC patients, although not statistically significant (*p* = 0.137). Biochemical data are presented in Table 2.

**Table 2:** CSF protein levels of inflammatory markers, total tau, and albumin index

Analyte	PD	MSA	PSP	Controls (SC)
CRP, mean (SD)^b^	5376.31 (4376.71)	8208.5 (3921.46)	8293.99 (4261.78)	6005.12 (4155.4)
IFN-γ, mean (SD)	1.35 (1.69)	1.33 (1.04)	2.11 (2.37)	3.09 (10.64)
IL-10, mean (SD)	4.57 (7.02)	4.71 (2.49)	6.18 (3.85)	5.66 (6.5)
IL-18, mean (SD)	47.85 (18.06)	53.25 (34.44)	50.52 (21.20)	45.30 (19.25)
IL-1β, mean (SD)^b^	2.82 (3.52)	3.58 (2.23)	4.96 (3.85)	2.60 (1.61)
IL-4, mean (SD)^b^	2.50 (2.21)	3.60 (2.22)	4.86 (3.90)	2.95 (1.90)
IL-6, mean (SD)^b^	53.41 (59.32)	86.98 (73.0)	102.53 (87.87)	61.74 (59.18)
TGF-β1, mean (SD)	1482.25 (407.20)	1435.18 (298.70)	1502.96 (416.43)	1518.89 (395.04)
TNF-α, mean (SD)^b^	79.78 (63.71)	106.80 (50.55)	127.21 (75.32)	87.65 (63.39)
Total tau protein, mean (SD)^b^	303.9 (213.9)	470.8 (242.6)	365.8 (187.5)	286.8 (196.4)
CSF protein, total, g/L, mean	0.53	0.52	0.54	0.42
CSF leukocytes, total, mean	2.91	1.50	2.16	2.60
Albumin index, mean	8.13	8.32	8.38	6.31

^a^Kruskal-Wallis *p* < 0.05, Bonferroni corrected

^b^*p* < 0.05 chi-square. All concentrations are in pg/ml, with the exception of CSF protein

### Diagnostic performance

**Fig. 2:**
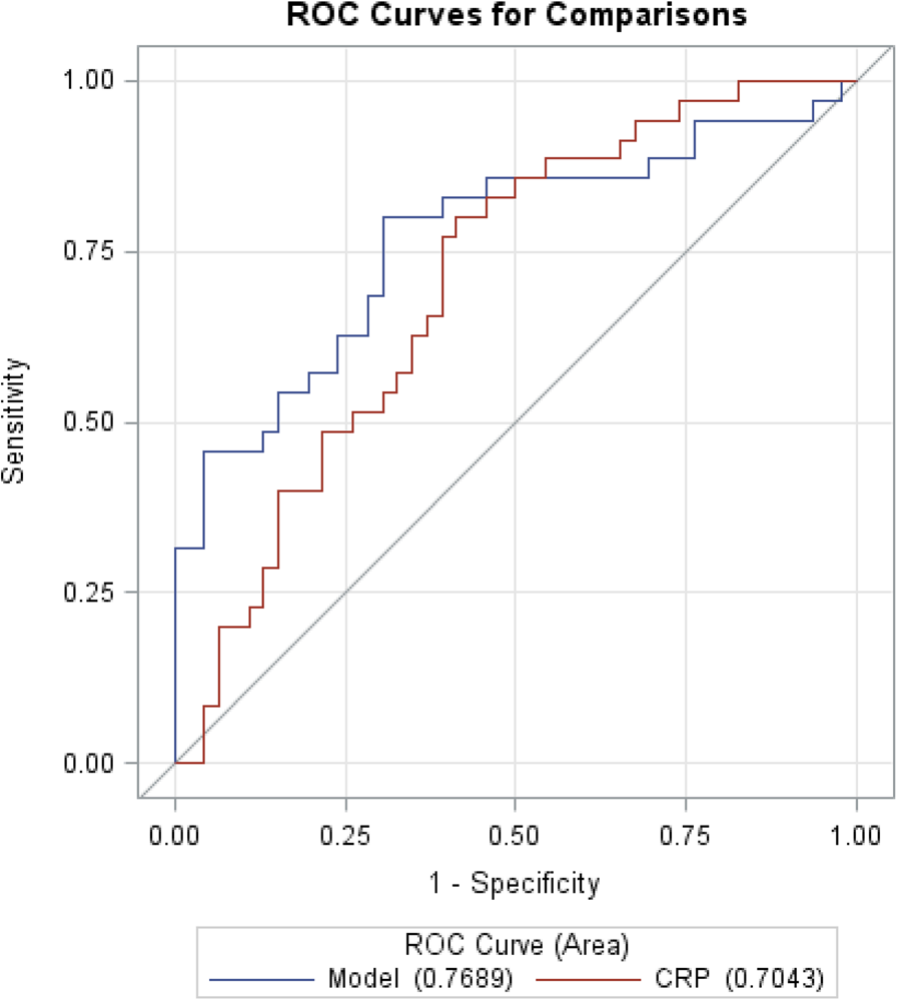
ROC curves discriminating MSA and PD patients based on the best subset of four analytes (CRP, TNF-α, IL-1β, IFN-y) and CRP

Investigated cytokines were evaluated with ROC curves assessing their individual ability to differentiate between the study groups. CRP was the best single analyte to discriminate between PD and MSA (AUC 0.70, *p* value 0.005, 95% CI 0.591–0.817). No other analyte achieved AUC > 0.70 (data not shown). CRP and a cytokine set (TNF-α, IL-1β, IFN-γ) was identified as the best subset to discriminate between MSA and PD (AUC = 0.77, *p* = 0.007, 95% CI 0.660–0.867). ROC curves (Fig. 2**)** including t-tau in the model yielded a best subset of CRP, TNF-α, IFN-γ, and t-tau (AUC 0.81, *p* < 0.001, 95% CI 0.713–0.901).

Il-1β (AUC 0.73, *p* value 0.019, 95% CI 0.624–0.837) was the best analyte to discriminate between PD and PSP followed by IL-4 (AUC 0.72, *p* value 0.012, 95% CI 0.611–0.830), CRP (AUC 0.70, *p* value 0.004, 95% CI 0.592–0.814), and TNF-α (AUC 0.70, *p* value 0.004, 95% CI 0.583–0.808). A subset of four analytes did not yield higher AUC between PD and PSP (data not shown).

## Discussion

We examined a selection of CSF cytokines among patients with PD or atypical Parkinson disorders, MSA, and PSP. To our knowledge, this is the first study of differences in CSF cytokine expression profiles comparing PD patients with those suffering from atypical Parkinsonism.

Our key findings included (1) increased concentrations of CRP and cytokines IL-1β, TNF-α, IL-6, and Il-4 among both MSA and PSP patients compared to PD patients, but not compared to SCs; (2) except for IL-4, no significant differences between PD and SCs; and (3) the best diagnostic discrimination between PD and MSA, evaluated by ROC curves, was found with a combination of analytes.

PD and MSA are both characterized by accumulation of abnormally aggregated alpha-synuclein (AS) in Lewy bodies and glial cytoplasmic inclusions, respectively [16]. Microglia is believed to be the key player in neuroinflammation in alpha-synucleinopathies. PSP, however, belongs to the tauopathies, where the protein Tau constitutes the neuropathological hallmark [17]. Although MSA and PSP present with differences in the neuropathology, both diseases are rapidly progressing atypical Parkinson disorders with severe symptomatology, and in our study, MSA and PSP exhibited similar patterns in CRP and cytokine expression, although PSP presented with the overall highest values (non-significant) of measured cytokines.

Activated microglia may take either an anti- or pro-inflammatory phenotype responsible for both protective and regenerative actions, as well as maintenance of chronic inflammation, possibly enhancing neurodegeneration [18]. TNF-α, IL-1β, and IL-6 are secreted by activated microglia and represent the pro-inflammatory pathway, and in our study, these cytokines were all significantly higher among MSA/PSP compared to those with PD. INF-γ stimulates the pro-inflammatory phenotype [18] and was not significantly different among groups. IL-10 represents the anti-inflammatory pathway and was significantly increased in PSP compared to PD, and likewise, IL-4 was also significantly increased in MSA/PSP compared to those with PD. IL-4 stimulates the anti-inflammatory phenotype [19]. Hence, our data may indicate an upregulation of microglia-derived cytokines in PSP and MSA, especially from the pro-inflammatory pathway, although the interaction between pathways is complex. CRP followed the same pattern. CRP is usually considered a liver-derived unspecific acute phase reactant but also expressed in the CNS, and one study found CRP to be synthesized in cell cultures of microglia [20].

One aim of this study was to investigate the biomarker potential focusing on the clinically difficult distinction between PD and MSA. PD and MSA often present with similar symptomatology in the initial phases of the diseases, and a valid biomarker would have profound clinical impact. We achieved the best diagnostic performance between MSA and PD patients with a subset of analytes (CRP, TNF-α, IL-1β, IFN-γ). The best subset of analytes was identified in a logistic regression model. Possible bias include overfitting, and given the relatively small sample size, additional studies are needed to validate.

Previously, CSF pro-inflammatory cytokines (IL-1β and IL-6 [7], TNF-α [8]) have been reported to be upregulated in PD patients compared to controls. Our results do not support the previous reports. A possible explanation may be related to differences in control groups. However, in a recent study investigating TNF-α, among others, in CSF, no significant differences were found between PD patients and a control patient group [21].

Confounding factors in our study included intake of anti-inflammatory drugs and comorbidity [22]. In addition, the distribution of gender, disease duration, and age was skewed across patient groups, and it is established that the systemic immune response changes with aging [23]. Hence, we adjusted analysis for age, gender, and use of anti-inflammatory drugs. We found no correlation between disease duration and cytokines, and PD patients presented with the longest disease duration. The age-adjusted Charlson comorbidity index did not differ significantly between patient groups.

Disease severity also must be addressed. We evaluated disease severity on the HY rating scale. The burden of pathology (neurodegeneration) reflects HY and disease severity in PD [14]. The median HY score differed (insignificantly) between PD (HY median = 2) and MSA and PSP (HY median = 3). However, the HY scale emphasizes on axial symptoms and postural instability. This may classify PSP and MSA with higher scores than is reflected by the burden of pathology. Disease severity and duration did not correlate within the patient groups.

The strengths of the study were the well-characterized patient cohort comprising for the orphan disorders of MSA and PSP high number of patients. We had information on important confounders and the CSF/plasma albumin ratio. The CSF/plasma albumin ratio represents a measure of possible blood-brain barrier leakage and cytokines from the peripheral circulation entering the CNS. We found no significant differences between the patient groups. Lastly, as pre-analytical factors may cause bias in biochemical studies due to differences in handling, sampling, or analyzing [24], it is worth noting that all samples in this study were handled similarly. No limit for blood contamination were set for this study. Blood contamination could possibly confound results. Hence, the first tubes were used for routine investigation only, and samples were centrifuged at 2000*g* after sampling which in a previous study [25] resulted in hemoglobin concentration below 200 ng/ml in all samples.

Our study also had some limitations. Firstly, all diagnoses were clinical, and since definite diagnosis requires neuropathology, misclassification is a possibility. However, the follow-up time was substantial in our cohort. Secondly, the control group included in this study was associated with limitations. Our reference group comprised of patients with acute onset headache where the CSF cytokine response, to our knowledge, is unknown. It is possible that acute onset headache would increase the CSF cytokine levels. Furthermore, data were available on regular use of anti-inflammatory drugs, but we did not have information on self-administered use of anti-inflammatory drugs, if any, prior to the admission, aiming to relief the headache of controls. Moreover, the controls were significantly younger than disease groups. Thirdly, we only investigated selected CSF cytokines, and we may have left out others of importance.

## Conclusion

In this well-described cohort of PD, MSA, and PSP patients, we analyzed a subset of inflammatory markers (CRP and eight cytokines) in the cerebrospinal fluid. Our study revealed a significant increase in pro-inflammatory and microglia-related cytokines (TNF-α, IL-1β, IL-6), CRP, and IL-4 in MSA and PSP patients compared to PD patients. No cytokine was a specific marker for parkinsonism, but TNF-α, CPP, IL-1β, IL-4, and IL-6 may be useful for early differentiation of atypical parkinsonism to PD.

## Additional file

Additional file 1: **Table S1.** Correlations between CRP and cytokine levels and clinical data. Correlations between analytes and age, disease duration, levodopa equivalents, Hoehn and Yahr stage, and total-Tau presented with Spearman’s rho and Bonferroni-corrected *p* values. (XLSX 10 kb)

## Abbreviations

APD: Atypical parkinsonism
CRP: C-reactive protein
CSF: Cerebrospinal fluid
IFN: Interferon
IL: Interleukin
MSA: Multiple system atrophy
PD: Parkinson’s disease
PSP: Progressive supranuclear palsy
TNF-α: Tumor necrosis factor alpha
